# Emergent Properties in Streptococcus mutans Biofilms Are Controlled through Adhesion Force Sensing by Initial Colonizers

**DOI:** 10.1128/mBio.01908-19

**Published:** 2019-09-10

**Authors:** Can Wang, Jiapeng Hou, Henny C. van der Mei, Henk J. Busscher, Yijin Ren

**Affiliations:** a University of Groningen and University Medical Center Groningen, W. J. Kolff Institute, Department of Orthodontics, Groningen, The Netherlands; b University of Groningen and University Medical Center Groningen, W. J. Kolff Institute, Department of Biomedical Engineering, Groningen, The Netherlands; Duke University School of Medicine

**Keywords:** OCT, atomic force microscopy, quorum sensing, regulation of gene expression, surface sensing

## Abstract

Bacterial adhesion is accompanied by altered gene expression, leading to “emergent” properties of biofilm bacteria that are alien to planktonic ones. With the aim of revealing the role of environmental adhesion forces in emergent biofilm properties, genes in Streptococcus mutans UA159 and a quorum-sensing-deficient mutant were identified that become expressed after adhesion to substratum surfaces. Using atomic force microscopy, adhesion forces of initial S. mutans colonizers on four different substrata were determined and related to gene expression. Adhesion forces upon initial contact were similarly low across different substrata, ranging between 0.2 and 1.2 nN regardless of the strain considered. Bond maturation required up to 21 s, depending on the strain and substratum surface involved, but stationary adhesion forces also were similar in the parent and in the mutant strain. However, stationary adhesion forces were largest on hydrophobic silicone rubber (19 to 20 nN), while being smallest on hydrophilic glass (3 to 4 nN). *brpA* gene expression in thin (34 to 48 μm) 5-h S. mutans UA159 biofilms was most sensitive to adhesion forces, while expression of *gbpB* and *comDE* expressions was weakly sensitive. *ftf*, *gtfB*, *vicR*, and *relA* expression was insensitive to adhesion forces. In thicker (98 to 151 μm) 24-h biofilms, adhesion-force-induced gene expression and emergent extracellular polymeric substance (EPS) production were limited to the first 20 to 30 μm above a substratum surface. In the quorum-sensing-deficient S. mutans, adhesion-force-controlled gene expression was absent in both 5- and 24-h biofilms. Thus, initial colonizers of substratum surfaces sense adhesion forces that externally trigger emergent biofilm properties over a limited distance above a substratum surface through quorum sensing.

## Abstract (precis)

A new concept in biofilm science is introduced: “adhesion force sensitivity of genes,” defining the degree up to which expression of different genes in adhering bacteria is controlled by the environmental adhesion forces they experience. Analysis of gene expression as a function of height in a biofilm showed that the information about the substratum surface to which initially adhering bacteria adhere is passed up to a biofilm height of 20 to 30 μm above a substratum surface, highlighting the importance and limitations of cell-to-cell communication in a biofilm. Bacteria in a biofilm mode of growth, as opposed to planktonic growth, are responsible for the great majority of human infections, predicted to become the number one cause of death in 2050. The concept of adhesion force sensitivity of genes provides better understanding of bacterial adaptation in biofilms, direly needed for the design of improved therapeutic measures that evade the recalcitrance of biofilm bacteria to antimicrobials.

## INTRODUCTION

Biofilms are surface-adhering and surface-adapted communities of microorganisms (1), in which adhesion to a substratum surface is the initial step. Two surfaces, including the surface of bacteria adhering on a substratum surface, can be attracted to each other by a combination of Lifshitz-van der Waals, electrostatic double-layer, and acid-base forces (2). The sum total of these forces is generally called the “adhesion force.” The environmental adhesion forces by which a bacterium adheres to a surface are orders of magnitude larger than the gravitational forces bacteria experience and give rise to nanoscopic deformation of the cell wall (3, 4). Cell wall deformation in its turn causes changes in lipid membrane surface tension that provides a stimulus for the environmentally triggered expression of a great number of genes in adhering bacteria (5) to facilitate their surface adaptation. This leads to new, so-called “emergent” properties of adhering bacteria in their biofilm mode growth (6). Emergent properties reflect bacterial surface adaptation and arise only after bacteria have adhered to a surface. According to their definition (6), emergent properties of bacteria in biofilm mode growth are alien to their planktonic counterparts and cannot even be predicted on the basis of the properties of planktonic bacteria. The most prominent, landmark emergent property of adhering bacteria is the production of an extracellular polymeric matrix in which biofilm bacteria protect themselves against host defenses (7) and antimicrobial agents (8, 9) and through which they enforce their bond with a substratum surface (10).

Adhesion-force-induced surface adaptation in adhering bacteria has been observed in Staphylococcus aureus biofilms for the *icaA* gene, regulating production of extracellular polymeric substances (EPS). However, adhesion-force-induced surface adaptation was not observed for the *cidA* gene, which is associated with cell lysis and extracellular DNA (eDNA) release (11). Also, nisin clearance in staphylococci through the two-component NsaRS intramembrane-located sensor NsaS and NsaAB efflux pump (12) was enhanced when staphylococci adhered more strongly to a substratum surface (13). Hitherto, adhesion force sensing and associated cell wall deformation have appeared as an appealing concept to explain what environmental stimulus externally triggers the development of emergent properties of bacteria in biofilm mode growth. Yet, there still are many questions to be addressed, most urgently concerning the range over which adhesion force sensing operates in a biofilm. Typically, biofilms are much thicker than the range of the adhesion forces extending from a substratum surface. Adhesion forces can yield an attraction that can be sensed up to maximally 0.5 μm into a biofilm (2, 3). The exact magnitude and range of an adhesion force depend on the hydrophobicity and charge properties of the bacterial cell and substratum surfaces. Compared with the thickness of a biofilm, the range over which adhesion forces operate is relatively short. This suggests that quorum sensing plays a role in spreading the “news” that initial colonizers in a biofilm have “landed” on a substratum surface exerting a specific adhesion force. However, this suggestion has never been confirmed. Furthermore, adhesion force sensing has never been confirmed in other species than staphylococci.

Adhesion to surfaces is a survival mechanism for streptococci in the oral cavity (14). Accordingly, Streptococcus mutans has the ability to adhere to oral hard and soft tissues, abiotic restorative dental materials, and other bacteria in the oral cavity (15). Frequently studied genes involved in S. mutans initial adhesion and biofilm formation are summarized in Table 1. Based on the definition of “emergent” properties as given by Flemming et al. (6) and literature description of gene functions, a hypothetical distinction is made between genes whose expression prepares planktonic bacteria for adhesion to a substratum surface and genes relevant for the development of emergent properties in adhering bacteria. For instance, genes that regulate synthesis of specific ligands of planktonic streptococci for optimal initial adhesion to saliva-coated surfaces, such as *ftf* and *gtfB* (16–19), are not considered to be involved in the development of emergent properties that arise by definition in already adhering bacteria. Also, genes regulating bacteriocin production, cell death, and chemical stress responses (*comDE*, *virR*, *gbpB*, and *relA*), although vital in biofilm formation, may not bear direct relevance to EPS production, enforcing strong adhesion of biofilm inhabitants to a substratum surface (20–22). Autoinducer 2 in the S. mutans *luxS* quorum-sensing system (see also Table 1) coordinates communication in S. mutans biofilms (23) and may be expected to impact the extension of adhesion-force-sensitive genetic programming into a mature biofilm, as adhesion forces can only be directly sensed by initial colonizers (4).

**TABLE 1:** Summary of genes involved in S. mutans UA159 initial adhesion and subsequent processes occurring during biofilm formation

Gene^*a*^	Function	Reference(s)
Genes relevant to prepare initial adhesion in planktonic S. mutans		
*ftf*	Catalysis of sucrose cleavage to synthesize fructan to promote initial adhesion to salivary films	16, 17
*gtfB*	Synthesis of water-insoluble glucans (α-1,3-linked) to promote initial adhesion to saliva-coated tooth surfaces and establishment of microcolonies in biofilm	18, 19
Genes relevant to develop emergent properties in adhering S. mutans		
*brpA*	Regulation of cell wall stress responses, biofilm cohesiveness, and biofilm formation	24, 33, 34
*comDE*	Persister cell formation, bacteriocin production	30
*vicR*	Synthesis of EPS matrix components, regulation of bacteriocin production and cell death	44, 45
*gbpB*	Regulation of sensitivity to antibiotics, osmotic and oxidative stresses, cell wall construction and maintenance, cell shape, hydrophobicity, and sucrose-dependent biofilm formation	28, 29
*relA*	Regulation of stringent response, acid tolerance, and biofilm formation	46, 47
*luxS*	Coordination of collective behaviors and cohesiveness in biofilms	48, 49

a A hypothetical distinction has been made with respect to genes relevant to prepare initial adhesion in planktonic streptococci and genes involved in the development of emergent properties in adhering bacteria.

In order to further advance the concept of adhesion-force-induced gene expression in relation to emergent biofilm properties, the aim of this article is first to identify genes involved in biofilm formation by S. mutans and an isogenic, quorum-sensing-deficient mutant whose expression is controlled by environmental adhesion forces. This would confirm the hypothetical distinctions made in Table 1 between genes preparing planktonic bacteria for adhesion to a substratum surface and genes relevant for the development of emergent properties in adhering bacteria. To this end, biofilms of S. mutans UA159 and its *ΔluxS* isogenic mutant were grown on four substratum surfaces with different hydrophobicities, and single-bacterial contact probe atomic force microscopy (AFM) was applied to measure the forces by which both strains adhere to each substratum surface. Gene expression was evaluated using RT-qPCR. Up- or downregulation of selected genes upon adhesion was related to the forces by which the streptococci adhere to yield a new concept of “adhesion force sensitivity of gene expression.” Uniquely, the extension of adhesion-force-induced genetic programming over the height of the biofilms above a substratum surface was investigated in cryosections of the biofilms taken at different heights above a substratum surface. Herewith it can be determined to what extent quorum sensing controls adhesion-force-induced gene expression in later biofilm inhabitants, residing further away from the substratum surface and not in direct contact with the substratum surface. Whiteness analyses of optical coherence tomography (OCT) images of biofilms was employed to support the conclusions regarding height-dependent gene expression taken from cryosections of the S. mutans biofilms.

## RESULTS

### Bacterial cell and substratum surface characteristics.

First, it was established that S. mutans UA159 and its isogenic mutant UA159 *ΔluxS* exhibited comparable cell surface characteristics, despite exchange of the *luxS* gene using an erythromycin resistance determinant (24). Hydrophobicity and charge are both important physicochemical bacterial cell surface characteristics involved in adhesion and in combination with comparable properties of the substratum surface define the magnitude of the adhesion forces (2). Cell surface hydrophobicity of bacteria is reflected among other characteristics by their removal from an aqueous phase by a hydrophobic ligand (see Fig. S1A in the supplemental material). Hydrophilic bacteria prefer to remain in the aqueous phase rather than being removed from it by adhesion to a hydrophobic ligand (25). Based on their equally low removal rates by hexadecane (*P* > 0.05, Mann-Whitney test), both strains can be classified as hydrophilic (Fig. S1B and C). In addition, streptococcal zeta potentials, reflecting surface charge, were slightly negative between −7 and −3 mV, with no significant differences between strains (*P* > 0.05, Mann-Whitney test). Like the hydrophobicity of the bacterial cell surfaces, the hydrophobicity of the substratum surfaces is also involved in bacterial adhesion and the forces by which bacteria adhere to a substratum surface. Water contact angles on substratum surfaces reflect the hydrophobicity of a material surface and were measured using the sessile drop technique (Fig. S1D). Water contact angles ranged from 11 to 103° for glass and silicone rubber surfaces, respectively, and differed significantly between all surfaces (*P* < 0.05, Mann-Whitney test). Also, hydrophobic, bacterial-grade and more hydrophilic, tissue-grade polystyrene surfaces (Fig. S1D) demonstrated a significant (*P* < 0.05, Mann-Whitney test) difference in water contact angles.

10.1128/mBio.01908-19.1FIG S1Bacterial cell and substratum surface characteristics involved in adhesion. (A) Microbial adhesion to hydrocarbons (MATH) in its kinetic mode (25) measures the removal of bacteria by a hydrophobic ligand from an aqueous phase as a function of vortexing time. Streptococci were suspended in buffer (1 mM CaCl_2_, 2 mM potassium phosphate, 50 mM KCl, pH 6.8) to an OD_600_ of between 0.4 and 0.6 (*A*_0_), and 150 μl hexadecane was added to the 3-ml bacterial suspension. The two-phase system was vortexed for 10 s and allowed to settle for 10 min, and the OD (*A_t_*) was measured. This procedure was repeated 6 more times, and results were plotted as log(*A_t_*/*A*_0_ × 100) against the vortexing time (*t*) to determine the rate of initial bacterial removal, *R*_0_ (min^−1^), from the aqueous phase (i.e., their hydrophobicity as by the kinetic MATH assay) according to
$R_{0}=\underset{t\to 0}{\text{lim}}\frac{d}{d_{t}}\text{log}\left(\frac{A_{t}}{A_{0}}\times 100\right)$Hydrophilic bacteria prefer to remain in the aqueous phase rather than be removed from it by adhesion to a hydrophobic ligand and have a low removal rate. (B) OD ratio log (*A_t_*/*A*_0_) × 100 of S. mutans UA159 and UA159 *ΔluxS* grown in BHI with 1% sucrose added as a function of vortexing time. Error bars, indicating SD over triplicate experiments with different streptococcal cultures, are smaller than the data points. (C) Hydrophobicities of bacterial cell and substratum surfaces, expressed by initial removal rates in MATH and water contact angles (WCA), respectively, together with the bacterial zeta potentials measured in buffer (3 × 10^8^ bacteria ml^−1^) using particulate microelectrophoresis (Zetasizer nano-ZS; Malvern Instruments, Worcestershire, United Kingdom) at 37°C. Bacterial cell surface characteristics are averages over triplicate experiments with different streptococcal cultures. “±” indicates SD values over three separate MATH assays, while in case of zeta potentials, “±” represents average SD values over a population of around 50 streptococci in a separate culture. (D) The hydrophobicities of the different substratum materials were determined through water contact angle measurements. Water contact angles were measured at 25°C using the sessile drop technique with a homemade contour monitor. Droplets of 1.5 to 2 μl ultrapure water were put on the different surfaces, and the contours of the droplet were measured between 5 and 10 s after placing a droplet, from which contact angles were subsequently calculated after gray value thresholding. Water contact angles are averages over triplicate experiments with different samples (with at least 3 spots chosen on each sample), with “±” indicating SD values. *PS, polystyrene. Superscript letters a to d in panel D indicate significant differences (*P* < 0.05, Mann-Whitney test) between four surfaces. Shown is spreading of sessile water droplets on the different substratum surfaces used. Note that part of a droplet reflects in the surface, enabling the determination of the exact position of the surface relative to which the contact angle must be calculated. Download FIG S1, TIF file, 2.0 MB.Copyright © 2019 Wang et al.2019Wang et al.This content is distributed under the terms of the Creative Commons Attribution 4.0 International license.

### Bacterial adhesion forces.

**FIG 1:**
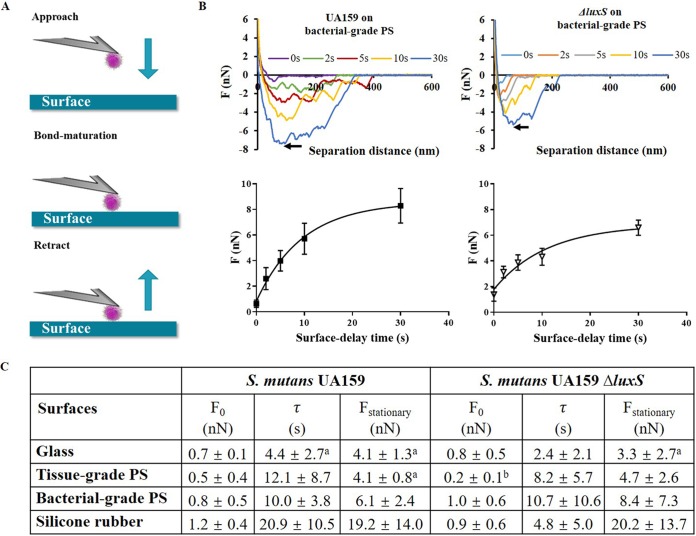
Bacterial adhesion force characteristics of both streptococcal strains on four substratum surfaces with different hydrophobicities. (A) Schematics of single-bacterial-contact probe atomic force microscopy. A bacterium is attached to a tipless AFM cantilever and brought to contact with a substratum surface, after which the cantilever is retracted following a surface delay that can be varied up to a maximum of 30 s. Upon retraction, the adhesion force by which the bacterium was attracted to the surface can be calculated from the cantilever bending. (B) Example of retraction force-distance curves taken after different surface delay times for S. mutans UA159 on a bacterial-grade polystyrene (PS) surface. (The arrow points to the force value, taken as the adhesion force.) Also included is a graph of streptococcal adhesion forces as a function of surface delay time for the parent strain and its quorum-sensing-deficient isogenic mutant. (C) Initial and stationary streptococcal adhesion forces *F*_0_ and *F*_stationary_, together with the characteristic bond maturation time constant τ on the different substratum surfaces. All data represent averages over 8 spots on 4 different surfaces of each substratum, measured with 4 different probes and bacteria from 4 different cultures, with ± signs representing standard deviation (SD) values over 32 measurements. Superscript letters in panel C indicate statistical significance as follows: a, statistically significant (*P* < 0.05, one-way ANOVA) differences from silicone rubber; b, statistically significant (*P* < 0.05, one-way ANOVA) differences between tissue-grade and bacterial-grade PS surfaces.

Streptococcal adhesion forces were measured on different substratum surfaces using single-bacterial-contact probe AFM (Fig. 1A). In single-bacterial-contact probe AFM, a bacterium attached to a flexible cantilever is brought into contact with a substratum surface and retracted after a specified time (the so-called “surface delay” or “bond maturation” time). Upon retraction, the cantilever bends until the bacterial bond with the substratum is disrupted. The force at which this occurs is subsequently calculated from the cantilever bending and recorded as the adhesion force of the bacterium to the substratum surface. Adhesion forces increased with increasing bond maturation time between the bacterium and a substratum surface. (See Fig. 1B for examples of force-distance curves taken after different bond maturation times for the parent strain and its isogenic, quorum-sensing-deficient mutant.) Adhesion forces as a function of bond maturation time followed an exponential increase (Fig. 1B). Accordingly, adhesion forces as a function of bond maturation time were fitted to equation 1

(1) $$F_{t}=F_{0}+(F_{\text{stationary}}-F_{0})[\text{exp}(-\frac{t}{\text{τ}})]$$

in which *t* denotes the surface delay time, *F*_0_ is the initial adhesion force at 0-s surface delay time, *F_t_* is the adhesion force after surface delay time *t*, and *F*_stationary_ indicates the stationary adhesion force, while τ is the characteristic time constant for bond maturation. Initial adhesion forces, *F*_0_ (Fig. 1C), were all in the sub-nN range on each substratum for the parent and the isogenic mutant strain (*P* > 0.05, one-way analysis of variance [ANOVA]). Bond maturation (compare τ values in Fig. 1C) occurred slower in the parent strain than in the isogenic mutant, especially on the silicone rubber. Like initial adhesion forces, stationary adhesion forces were similar in the parent strain and the isogenic mutant (*P* > 0.05, one-way ANOVA) when measured on the same material and increased for both strains with increasing hydrophobicity of the substratum surfaces. The difference between the two extremes in hydrophobicity on the glass and silicone rubber surfaces was significant within each strain (*P* < 0.05, one-way ANOVA).

### Streptococcal biofilm growth and gene expression.

Streptococcal biofilms were grown, and their thicknesses were evaluated using optical coherence tomography (OCT) (see Fig. S2A in the supplemental material). Twenty-four-hour biofilms were all significantly (*P* < 0.05, Mann-Whitney test) thicker than 5-h biofilms. Five-hour biofilms showed thicknesses ranging from 34 to 48 μm for S. mutans UA159 and from 26 to 34 μm for its isogenic mutant, UA159 Δ*luxS* (Fig. S2B). Comparison within each substratum surface showed these differences between strains to be not statistically significant (*P* > 0.05, Mann-Whitney test).

10.1128/mBio.01908-19.2 FIG S2 Examples of OCT images of S. mutans UA159 and UA159 Δ*luxS* biofilms and their thicknesses. (A) OCT cross-sectional images of 5- and 24-h streptococcal biofilms on the different substratum surfaces involved in this study. The scale bar represents 100 μm. (B) Thicknesses (μm) of 5- and 24-h streptococcal biofilms on the different substratum surfaces, determined from the OCT images after Otsu thresholding (42). All 24-h biofilms are significantly (*P* < 0.05, Mann-Whitney test) thicker than corresponding 5-h biofilms. “±” indicates SD over different experiments with separately cultured bacteria (*n* = 3). Download FIG S2, TIF file, 1.1 MB. Copyright © 2019 Wang et al. 2019 Wang et al. This content is distributed under the terms of the Creative Commons Attribution 4.0 International license.

**FIG 2:**
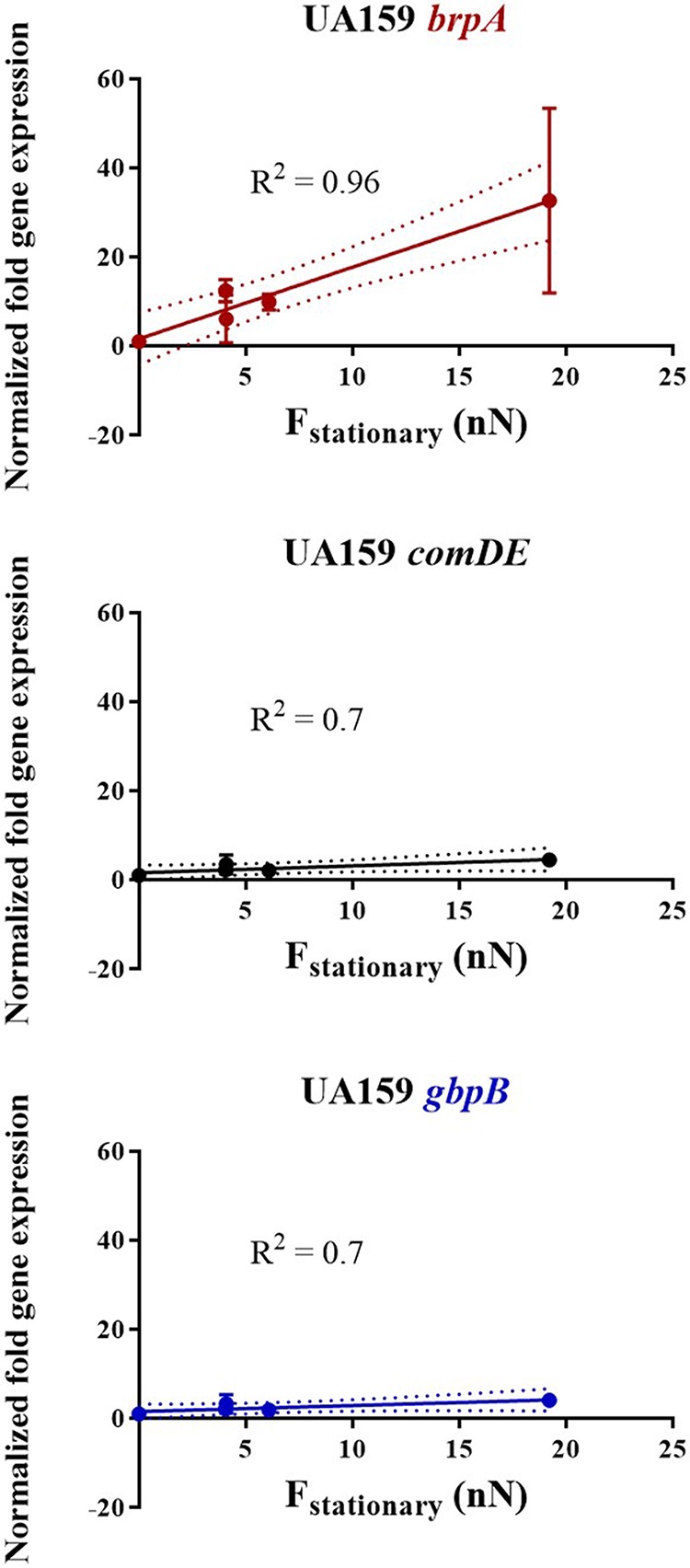
Normalized fold gene expression with significant relationships to adhesion forces in S. mutans UA159 as a function of the stationary adhesion force to different substratum surfaces over the entire height of 5-h biofilms. Error bars denote SD values in fold gene expression over triplicate experiments, while the solid lines represent assumed linear relationships through the data points, with the correlation coefficient *R*^2^ as presented. Dotted lines represent 95% confidence intervals.

Next, gene expression was evaluated in all streptococcal biofilms and normalized with respect to gene expression in planktonic streptococci of the corresponding strain (see Fig. S3A in the supplemental material). (Examples of amplification and melting curves are presented in Fig. S4 in the supplemental material.) An example of a heat map for the different genes expressed on different substrata for S. mutans UA159 is given in Fig. S3B. Note that all gene expression was also normalized with respect to expression of the internal control gene 16S rRNA, and thus, different bacterial numbers will not affect the evaluation of gene expression. Gene expression as normalized with respect to planktonic streptococci varied in each strain on the different substratum surfaces, in both 5- and 24-h biofilms (Fig. S3C and D, respectively). Subsequently, normalized gene expression on different substrata was plotted as a function of the environmental adhesion forces experienced by each of the two streptococcal strains (Fig. 2; see Fig. S5 and Fig. S6 in the supplemental material). In the parent strain, significant linear relationships (correlation coefficients of 0.7 or higher [Fig. 2]) were observed for three (*brpA*, *comDE*, and *gbpB*) out of the seven genes evaluated in 5-h biofilms. However, in 5-h biofilms of the isogenic quorum-sensing-deficient mutant, none of the genes showed such linear relationships (correlation coefficients less than 0.7) and gene expression was considered not to be governed by adhesion forces. In cases where correlation coefficients were 0.7 or higher, the slopes in the graphs representing gene expression versus adhesion force can be interpreted as the sensitivity of a given gene to adhesion forces (Table 2). This renders expressions of *comDE* and *gbpB* genes as weakly sensitive to environmental adhesion forces, while externally triggered expression of *brpA* was strongly adhesion force sensitive in the parent strain. Note that when evaluated over the entire thickness of the 3- to 4-fold-thicker 24-h biofilms, none of the genes showed adhesion-force-induced expression (Fig. S6), regardless of the strain involved.

**TABLE 2:** Adhesion force sensitivity of different genes over the entire height of 5- and 24-h S. mutans UA159 and UA159 Δ*luxS* biofilms^*a*^

Gene	S. mutans UA159				S. mutans UA159 *ΔluxS*			
	Adhesion force sensitivity (nN^−1^)		*R*^2^		Adhesion force sensitivity (nN^−1^)		*R*^2^	
	5 h	24 h	5 h	24 h	5 h	24 h	5 h	24 h
*ftf*			0.3	0.4			<0.1	0.4
*gtfB*			0.1	0.5			0.3	0.1
*brpA*	**1.6**		**0.96**	<0.1			0.6	0.2
*comDE*	**0.2**		**0.7**	0.2			0.1	0.2
*vicR*			0.3	0.1			<0.1	0.2
*gbpB*	**0.1**		**0.7**	0.1			0.6	0.2
*relA*			0.3	<0.1			0.3	<0.1

a Linear relationships between gene expression and stationary adhesion force with a correlation coefficient of less than 0.7 were considered insignificant, and no sensitivity values were derived. Data in boldface are considered significant.

10.1128/mBio.01908-19.3 FIG S3 Biofilm gene expression in S. mutans UA159 and UA159 Δ*luxS* biofilms, grown on four different substratum surfaces, after 5 and 24 h. (A) Gene expression in biofilms was normalized to planktonic streptococci in each of the two strains used. (B) Heat map of gene expression in 5-h biofilms of S. mutans UA159 on the different substratum surfaces. The pseudocolor scale represents the level of expression of the respective genes. (C) Fold expression of selected genes in 5-h streptococcal biofilms on different substratum surfaces, normalized with respect to planktonic streptococci in each of the two strains used. (D) Same as panel C, now for 24-h biofilms. Download FIG S3, TIF file, 2.4 MB. Copyright © 2019 Wang et al. 2019 Wang et al. This content is distributed under the terms of the Creative Commons Attribution 4.0 International license.

10.1128/mBio.01908-19.4 FIG S4 Example of *brpA* amplification and melting curves in 5-h biofilms of both streptococcal strains on silicone rubber surfaces (results from duplicate wells). The green line indicates background fluorescence. Download FIG S4, TIF file, 0.7 MB. Copyright © 2019 Wang et al. 2019 Wang et al. This content is distributed under the terms of the Creative Commons Attribution 4.0 International license.

10.1128/mBio.01908-19.5 FIG S5 Normalized fold gene expression with insignificant relationships with adhesion forces in S. mutans UA159 and its Δ*luxS* isogenic mutant as a function of the stationary adhesion force to different substratum surfaces over the entire height of 5-h biofilms. Error bars denote SD values in fold gene expression over triplicate experiments, while the solid lines represent assumed linear relationships through the data points, with the correlation coefficient *R*^2^ as presented. Dotted lines represent 95% confidence intervals. Download FIG S5, TIF file, 2.5 MB. Copyright © 2019 Wang et al. 2019 Wang et al. This content is distributed under the terms of the Creative Commons Attribution 4.0 International license.

10.1128/mBio.01908-19.6 FIG S6 Normalized fold gene expression with insignificant relationships to adhesion forces in S. mutans UA159 and its Δ*luxS* isogenic mutant as a function of the stationary adhesion force to different substratum surfaces over the entire height of 24-h biofilms. Error bars denote SD values in fold gene expression over triplicate experiments, while the solid lines represent assumed linear relationships through the data points, with the correlation coefficient *R*^2^ as presented. Dotted lines represent 95% confidence intervals. Download FIG S6, TIF file, 2.1 MB. Copyright © 2019 Wang et al. 2019 Wang et al. This content is distributed under the terms of the Creative Commons Attribution 4.0 International license.

### Extension of adhesion-force-induced gene expression into a biofilm.

**FIG 3:**
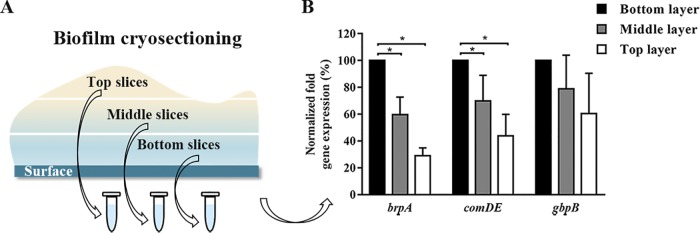
Gene expression in different layers of 24-h S. mutans UA159 biofilm on a silicone rubber surface. (A) Schematics of biofilm cryosectioning and gene expression in three biofilm slices taken at different heights in the biofilm above the substratum surface. (B) Percentage of normalized (with respect to the internal 16S rRNA control) adhesion-force-induced expression of selected genes at different heights above a silicone rubber surface in 24-h S. mutans UA159 biofilm, expressed relative to gene expression in the bottom layer of the biofilm closest to the substratum surface, set at 100%. Error bars denote SD values over triplicate experiments. *, statistically different at *P* < 0.05 by one-way ANOVA.

In order to determine how far adhesion-force-induced gene expression extended into a biofilm, levels of gene expression at different heights above a substratum surface (Fig. 3A) were evaluated in cryosectioned slices with a thickness of approximately 30 μm. Silicone rubber was chosen, because in 5-h biofilms grown on silicone rubber, most genes studied were expressed most strongly (Fig. S3C). Since 5 h biofilms were too thin for sectioning, sectioning was only done on 24-h biofilms. Setting gene expression normalized with respect to the internal 16S rRNA control and closest to the substratum surface at 100%, it can be seen in Fig. 3B that the adhesion-force-induced expression of *brpA* and *comDE* was significantly decreased (*P* < 0.05, one-way ANOVA) in the middle and top layers of the biofilm compared to the 30-μm bottom layers, decreasing to 30 to 70% in the top layer of the biofilm, depending on the gene considered.

### Extension of water- and EPS-filled pockets in streptococcal biofilms.

**FIG 4:**
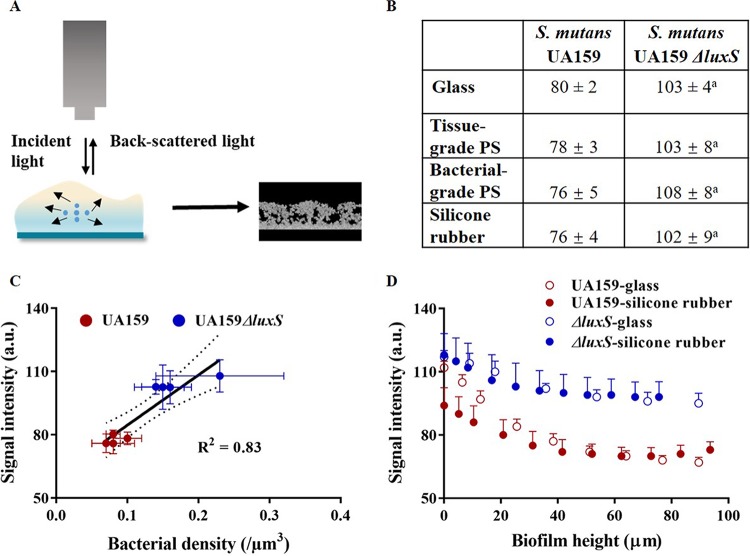
Analysis of OCT images of 24-h S. mutans UA159 and UA159 Δ*luxS* biofilms. (A) Schematics of signal intensity development by back-scattered light in OCT: based on an artificial whiteness scale, bacteria yield white regions (high signal intensity) due to back-scattering, while water- and soluble EPS-filled pockets do not back-scatter light and appear as black regions (low signal intensity). (B) Average signal intensity over an entire biofilm in 24-h streptococcal biofilms on the four different substratum surfaces. The superscript letter a in panel B indicates significant difference between S. mutans UA159 and UA159 Δ*luxS* (*P* < 0.05, Mann-Whitney test). (C) Average signal intensity over an entire biofilm as a function of the volumetric bacterial density for 24-h streptococcal biofilms of both strains on the four different substratum surfaces. Dotted lines represent 95% confidence intervals. (D) Local signal intensity in OCT images of 24-h streptococcal biofilms on glass and silicone rubber as a function of the biofilm height above the substratum surface. There are no statistically significant (*P* > 0.05, Mann-Whitney test) differences at corresponding heights for the mutant strain on hydrophobic silicone rubber and hydrophilic glass, while for the parent strain, signal intensities are lower on silicone rubber than on hydrophilic glass up to a thickness of 20 to 25 μm. Error bars indicate SD over different experiments with separately cultured bacteria (*n* = 3).

OCT imaging of biofilms allows comparison of biofilm regions with different levels of back-scattering of incident light that can be associated with bacteria, insoluble EPS, and water- and soluble EPS-filled pockets (26). (See Fig. 4A for schematics.) Since bacteria are much larger than insoluble EPS molecules, most back-scattered light originates from bacterial presence, as confirmed recently for a wide variety of bacterial strains and species by a relationship between signal intensities in OCT images and volumetric bacterial densities (26). Using an artificial whiteness scale (white representing the highest signal intensity of back-scattered light), the average whiteness in images of 24-h S. mutans UA159 biofilms was significantly (*P* < 0.05, Mann-Whitney test) lower on all substratum surfaces than in biofilm images of S. mutans UA159 Δ*luxS* (Fig. 4B). This suggests that the great majority of individual bacteria in S. mutans UA159 biofilms were triggered to produce soluble EPS, while biofilm images of quorum-sensing-deficient S. mutans UA159 Δ*luxS* appeared much whiter in the absence of water- and soluble EPS-filled pockets. As a consequence of differential soluble EPS production, the volumetric density of bacteria in streptococcal biofilms (i.e., the number of bacteria per unit of biofilm volume, determined by enumeration of the number of bacteria after biofilm dispersal from a defined substratum surface area, and subsequently divided by the biofilm volume) was lower (*P* < 0.05, Mann-Whitney test) for the parent strain than for the quorum-sensing-deficient mutant and related linearly to the average signal intensity in OCT images (Fig. 4C). Analysis of the local signal intensity in OCT images as a function of height above the substratum surfaces demonstrates that signal intensities of the S. mutans UA159 Δ*luxS* images (Fig. 4D) varied in a nearly identical fashion above both surfaces. However, in biofilm images of the parent strain, local signal intensities as a function of height above the surface suggest more extensive (*P* < 0.05, Student's *t* test) soluble EPS production on the hydrophobic silicone rubber surface than on the hydrophilic glass surface up to a height of 20 to 25 μm above the surfaces.

## DISCUSSION

S. mutans is an avid sugar consumer in the oral cavity, allowing it to produce acids that make it one of the world’s most widespread pathogens, responsible for the decalcification of oral hard tissues. For its survival in the oral cavity, S. mutans needs to adhere (14). Once adhering, S. mutans enforces its adhesion to oral surfaces through the production of EPS (27) as a landmark, emergent biofilm property. In this article, we identified *gbpB*, *brpA*, and *comDE* as genes that became more strongly expressed upon adhesion of S. mutans UA159, compared with *ftf*, *gtfB*, *vicR*, and *relA*. This confirms our hypothetical distinction (Table 1) of *ftf* and *gtfB* genes being more relevant for the preparation of planktonic streptococci for their initial adhesion to surfaces. Also, it justifies the classification of the *gbpB*, *brpA*, and *comDE* genes as more relevant for the development of emergent properties in adhering streptococci. The *vicR* and *relA* genes play roles with respect to diverse processes occurring during biofilm formation (Table 1), but these are not exclusively involved in directly enforcing the initial adhesion of S. mutans to oral surfaces.

Based on the differential expression of the *gbpB*, *brpA*, and *comDE* genes in streptococci adhering on different substratum surfaces and relating it to the adhesion forces experienced by adhering bacteria, a new concept of “adhesion force sensitivity of gene expression” is introduced. Adhesion force sensitivity reflects whether expression of a gene is more or less strongly influenced by the adhesion force sensed by bacteria upon their adhesion to a substratum surface. Among the three genes identified, *gbpB* had the weakest adhesion force sensitivity. However, *gbpB* is not only involved in enforcing initial streptococcal adhesion but also possesses an array of other pivotal functions in biofilm formation (Table 1) (28, 29). *comDE* is also weakly adhesion force sensitive and also possesses other functions than enforcing initial adhesion, including persister cell formation (30). However, persister cell formation usually involves bacteria closely associated with a substratum surface (31), and hence the weak control of adhesion forces over *comDE* expression as determined over the entire height of a biofilm is not surprising. Moreover, these weakly adhesion-force-sensitive genes as identified in this study have also been found to be upregulated in biofilm detached cells (32). Detachment is an important mechanism for bacterial survival, since it protects the biofilm from overpopulation, which is opposite from enforcing initial adhesion. Expression of *brpA* was by far several fold more sensitive to adhesion forces than *gbpB* and *comDE*, and its role in biofilm formation has been forcefully emphasized in the literature (24, 33, 34).

When averaged over the entire height of relatively thin, 5-h biofilms of S. mutans UA159, biofilms demonstrated adhesion-force-controlled gene expression, but this was not observed in thicker, 24 h biofilms (Table 2). In order to study the biofilm height above a substratum surface over which initially adhering streptococci in direct contact with a substratum surface can signal the news of being in an adhering state on a specific surface, 24-h biofilms on silicone rubber were sliced (Fig. 3A). Biofilm slices taken at different heights were examined for expression of the three adhesion-force-sensitive genes identified. In 24-h biofilms, slices taken closest to the substratum surface demonstrated higher expression of the three adhesion-force-sensitive genes than slices of biofilm taken more distant from the surface (Fig. 3B). Thus, adhesion-force-induced gene expression extended over at least half of the biofilm height above a surface, which represents a considerably larger distance than that over which adhesion forces arising from the substratum surface can range (2, 3). In addition to this, most bacteria in a biofilm have never visited a substratum surface (35). This implies that quorum sensing must be responsible for the extension of adhesion-force-induced gene expression in biofilms. This conclusion is supported by the observation that adhesion-force-induced gene expression of quorum-sensing-deficient S. mutans UA159 Δ*luxS* was fully absent in both 5- and 24-h-old biofilms (Table 2).

Moreover, in quorum-sensing-deficient S. mutans UA159 Δ*luxS*, EPS production reflected by local back-scattered light intensities (Fig. 4D) showed identical distributions of soluble EPS over the height of biofilms on silicone rubber and glass (Fig. 4D). Alternatively, in biofilms of S. mutans UA159 with the ability of quorum sensing, soluble EPS production on hydrophobic silicone rubber was higher than on hydrophilic glass up to a distance of around 20 to 25 μm above the substratum surface. Thus, it can be concluded based on height-dependent gene expression and local EPS production that adhesion-force-induced expression of genes extends into a biofilm through quorum sensing over a height limited to 20 to 30 μm above the substratum surface, beyond which autoinducer concentrations become below their threshold concentrations required to invoke a response. “Calling” distances over which bacteria can communicate through quorum sensing have been reported between 5 μm (36) and 200 μm (37), which indicates that our estimate of 20 to 30 μm as the calling distance in streptococcal biofilms is reasonable.

In summary, this work extends our understanding of emergent properties in streptococcal biofilms and the role of quorum sensing herein. Environmental adhesion forces have been identified to externally control expression of genes that are directly involved in the development of emergent biofilm properties in adhering S. mutans, leading to a new concept of “adhesion-force-induced gene expression in adhering bacteria.” *brpA* was the most adhesion-force-sensitive gene, as well as the most strongly expressed gene in adhering streptococci. Extension of its expression decreased with height above the substratum surface. Adhesion-force-induced gene expression was fully absent in a quorum-sensing-deficient isogenic streptococcal mutant. The concept of adhesion-force-induced gene expression and its extension through a biofilm through quorum-sensing mechanisms advance our understanding of why biofilms of the same strain or species may possess different properties when grown on different substrata, which is relevant in all environmental, industrial, and biomedical applications where biofilms develop.

## MATERIALS AND METHODS

### Bacterial strains, growth conditions, and harvesting.

S. mutans UA159 and UA159 *ΔluxS* were cultured at 37°C in 5% CO_2_ on blood agar for 24 h. One colony was inoculated in 10 ml brain heart infusion (BHI) broth (Oxoid, Basingstoke, United Kingdom) with 1% (wt/vol) sucrose added at 37°C in 5% CO_2_ for 24 h. These precultures were used to inoculate the main cultures (1:20 dilution), which were grown for 16 h. For S. mutans UA159 *ΔluxS*, 30 μg/ml erythromycin was added to both precultures and main cultures. Bacteria were harvested by centrifugation (Beckman J2-MC centrifuge; Beckman Coulter, Inc., Pasadena, CA, USA) for 5 min at 5,000 × *g* and washed twice with freshly made buffer (1 mM CaCl_2_, 2 mM potassium phosphate, 50 mM KCl, pH 6.8) and resuspended in buffer. In order to break streptococcal chains, bacterial suspensions were sonicated 3 times for 10 s each with 30-s intervals at 30 W (Vibra cell model 375; Sonics and Materials, Inc., Danbury, CT, USA), while cooling in an ice-water bath. The bacterial suspensions were diluted in buffer to a concentration appropriate for the respective experiments, as determined by enumeration in a Bürker-Türk counting chamber or measurement of the optical density at 600 nm (OD_600_).

### Bacterial cell surface characterization.

Microbial adhesion to hydrocarbons (MATH) (Fig. S1) was carried out in its kinetic mode (25) to reveal possible differences in adhesive cell surface properties between S. mutans UA159 and UA159 *ΔluxS*. To this end, streptococci were suspended in buffer to an OD_600_ of between 0.4 and 0.6 (*A*_0_), and 150 μl hexadecane was added to 3 ml of bacterial suspension. The two-phase system was vortexed for 10 s and allowed to settle for 10 min. The optical density (*A_t_*) was measured, this procedure was repeated 6 more times, and the results were plotted as log(*A_t_*/*A*_0_ × 100) against the vortexing time (*t*) to determine the rate of initial bacterial removal, *R*_0_ (min^−1^), from the aqueous phase (i.e., their hydrophobicity) as by the kinetic MATH assay, according to equation 2:

(2) $$R_{0}=\underset{t\to 0}{\text{lim}}\frac{d}{d_{t}}\text{log}\left(\frac{A_{t}}{A_{0}}\times 100\right)$$

Zeta potentials of both S. mutans strains (3 × 10^8^ ml^−1^) were determined in buffer by particulate microelectrophoresis (Zetasizer nano-ZS; Malvern Instruments, Worcestershire, United Kingdom) at 37°C. All bacterial cell surface characterizations were done in triplicate with different bacterial cultures, and data are presented as averages ± standard deviations (SD) of the mean.

### Substratum materials and characterization.

Four different substratum materials were used in this study: glass (Thermo Scientific, Braunschweig, Germany), bacterial-grade polystyrene (Greiner Bio-One GmbH, Frickenhausen, Germany), tissue-grade polystyrene (Greiner Bio-One GmbH), and medical-grade silicone rubber (ATOS Medical B.V., Zoetermeer, The Netherlands). Polystyrene is a hydrophobic material, mostly applied in microbiology for well plates to keep bacteria in suspension. Therefore, the company also advocates it for use as “suspension culture plates” made of hydrophobic “bacterial-grade” polystyrene. In cell biology, a hydrophilically modified type of polystyrene is preferred, since cells grow on surfaces. These plates are called “tissue culture plates” made of relatively hydrophilic “tissue-grade” polystyrene. All materials were made to fit into a 24-well plate, allowing samples with a surface area of 1 cm^2^. Polystyrene surfaces were used as received, while glass and silicone rubber surfaces were cleaned first with 2% RBS (Rue Bollinckx, Brussels, Belgium) under sonication and rinsed with warm tap water, sterilized in ethanol (96%), and finally washed with sterilized buffer.

The hydrophobicities of the different substratum materials were determined through water contact angle measurements. Water contact angles were measured at 25°C using the sessile drop technique with a homemade contour monitor. Droplets of 1.5 to 2 μl ultrapure water were put on the different surfaces, and the contours of the droplet were measured between 5 and 10 s after placing a droplet, from which contact angles were subsequently calculated after gray value thresholding. Contact angles were measured in triplicate on each of the four materials.

### Adhesion force measurement.

Single-bacterial-contact probes were prepared by attaching streptococci to a tipless cantilever (NP-O10; Bruker AFM Probes, Camarillo, CA, USA) via electrostatic interaction with poly-l-lysine (PLL) (molecular weight, 70,000 to 150,000; Sigma-Aldrich, St. Louis, MO, USA) adsorbed to the cantilever using a micromanipulator (Narishige Groups, Tokyo, Japan). Cantilevers were calibrated using the thermal method (38), yielding spring constants in the range of 0.03 to 0.12 N/m. Briefly, the far end of a tipless cantilever was dipped in a droplet of PLL for 1 min and dried in air for 2 min, followed by 2 min of immersion in a droplet of bacterial suspension (3 × 10^7^ ml^−1^ in buffer) to allow one bacterium to adhere to the cantilever. Attachment to the PLL-coated cantilever did not affect the viability of the bacteria (39, 40). Freshly prepared bacterial probes were directly used for adhesion force measurements. Adhesion force measurements (Fig. 1A) were performed at room temperature in buffer using a Dimension 3100 system (Nanoscope V; Digital Instruments, Woodbury, NY, USA). For each bacterial probe, force-distance curves were measured with 0, 2, 5, 10, and 30 s of surface delay at a 5-nN trigger threshold. In order to verify whether a measurement series had disrupted bacterial integrity, five force-distance curves at a loading force of 5 nN and surface delay of 0 s were measured at the beginning and end of each experiment on glass. When the adhesion forces measured differed more than 1 nN from the beginning to the end of an experiment, data were discarded and the probe was replaced by a new one.

### Biofilm formation.

Silicone rubber and glass samples were put in 24-well plates of either bacterial or tissue grade, and initial bacterial adhesion was allowed by adding 1 ml of streptococcal suspension (3 × 10^8^ ml^−1^) in buffer to each well under static conditions for 2 h at 37°C under 5% CO_2_. In addition, initial adhesion was allowed on the bottom of 24-well plates of either bacterial or tissue grade. After 2 h, the bacterial suspension was removed, and each well was carefully washed once with 1 ml buffer, after which 1 ml BHI with 1% sucrose (wt/vol) was added to each well to allow biofilm growth under a static condition in 5% CO_2_ at 37°C. After 5 or 24 h of growth, biofilms were carefully washed with buffer and then imaged with OCT (Thorlabs Ganymede, Newton, NJ, USA) to determine their thickness and whiteness distribution over the biofilm height above the substratum surface. Then streptococcal biofilms were carefully scraped off the surfaces and resuspended in buffer for gene expression or for bacterial enumeration in a Bürker-Türk counting chamber as described above in order to calculate volumetric bacterial densities in the biofilm, defined as the number of bacteria divided by the volume they occupy in a biofilm. Alternatively, intact biofilms were embedded in Tissue-Tek OCT compound (Sakura Finetek USA, Inc., Torrance, CA, USA) and stored at –80°C for later cryosectioning.

### Gene expression of planktonic and biofilm-grown bacteria.

**(i) Gene expression in planktonic and resuspended biofilms.** Planktonic as well as resuspended biofilm-grown streptococci were centrifuged at 6,500 × *g* for 5 min, the supernatant was removed, and pellets were stored at –80°C until RNA isolation. In order to prevent possible alterations in gene expression during sample collection, resuspension, centrifugation, and freeze storage were done as fast as possible (less than 45 min). Total RNA was isolated using RiboPure bacterial kit (Ambion, Invitrogen, Foster City, CA) according to the manufacturer’s instructions. Traces of genomic DNA were removed using the DNAfree kit (Ambion, Applied Biosystems, Foster City, CA). The amount and quality of extracted RNA were based on the 260/280-nm ratio measured using a NanoDrop ND-1000 (NanoDrop Technologies LLC, Thermo Fisher Scientific, Wilmington, DE). A ratio of around 2.0% ± 10% was accepted as ‘‘pure” for RNA. A mixture of 200 ng RNA, 4 μl 5 × iScript reaction mixture, and 1 μl iScript reverse transcriptase, in a total volume of 20 μl (Iscript; Bio-Rad, Hercules, CA), was used for cDNA synthesis according to the manufacturer’s instructions. Real-time reverse transcription-quantitative PCR (RT-qPCR) was performed in a 384-well plate (HSP-3905; Bio-Rad Laboratories, Foster City, CA, USA) with the primer sets for the selected genes (see Table S1 in the supplemental material). The following thermal conditions were used for all RT-qPCRs: 95°C for 3 min and 39 cycles of 95°C for 10 s and 59°C for 30 s. The mRNA levels were quantified in relation to endogenous control gene coding for 16S rRNA. Gene expression levels in the biofilms were normalized to planktonic S. mutans UA159. Gene expression was assessed in triplicate experiments with separately grown cultures.

10.1128/mBio.01908-19.7 TABLE S1 Primer sequences for RT-qPCR used in this study. Download Table S1, DOCX file, 0.05 MB. Copyright © 2019 Wang et al. 2019 Wang et al. This content is distributed under the terms of the Creative Commons Attribution 4.0 International license.

**(ii) Gene expression in biofilm slices as a function of biofilm height above a substratum surface.** Twenty-four-hour biofilms grown on silicone rubber surfaces were washed with freshly made buffer and removed from their 24-well plates. Tissue-Tek OCT compound (Sakura Finetek USA, Inc., Torrance, CA, USA) was applied to the biofilm surface, and thus embedded biofilms were subsequently stored at –80°C. Embedded biofilms were sliced using a cryostat into 10-μm-thick slices taken parallel to the substratum surface. The top, middle, and bottom slices of biofilm (6 slices of 10 μm of the biofilm) were collected separately in 1.5-ml tubes and stored at –80°C for further RNA isolation and analysis of the expression of selected genes, as described above. Finally, gene expression was normalized with respect to gene expression in the layer adjacent to the substratum surface (i.e., the bottom slices).

### OCT imaging.

Biofilms were imaged using an OCT Ganymede II (Thorlabs Ganymede, Newton, NJ, USA) with a 930-nm center wavelength white light beam and a Thorlabs LSM03 objective scan lens, providing a maximum scan area of 100 mm^2^. The imaging frequency was 30 kHz, with a sensitivity of 101 dB, and the refractive index of biofilm was set as 1.33, equal to the one of water. Two-dimensional (2D) images had fixed 5,000 pixels with variable pixel size, depending on magnification in the horizontal direction, while containing a variable number of pixels with a 2.68-μm pixel size in the vertical direction. Images were created by the OCT software (ThorImage OCT 4.1) using 32-bit data, and signal intensities of back-scattered light were reflected by a whiteness distribution in OCT images (41). Biofilm thickness was subsequently determined from the OCT images after Otsu thresholding (42). To eliminate the influence of autoscaling by the instrument on signal intensities of back-scattered light, rescaling was applied (26, 43). Rescaled signal intensities have been demonstrated to reflect the absence or presence of water- and EPS-filled pockets in a biofilm and relate to the volumetric bacterial density in biofilms (26, 43).

### Statistical analysis.

GraphPad Prism, version 7 (San Diego, CA), was employed for statistical analysis. Significance among groups was assessed by one-way analysis of variance (ANOVA) followed by Dunn’s multiple-comparison test. Alternatively, the Mann-Whitney test was used to compare two sets of data at a time. For comparison of OCT signal intensities at different biofilm heights, Student's *t* test was applied. Significance was adapted at *P* < 0.05.
