# Supplementary figures and images for: Emergent Properties in Streptococcus mutans Biofilms Are Controlled through Adhesion Force Sensing by Initial Colonizers
Source: mBio. 2019 Sep 10;10(5):e01908-19. doi: 10.1128/mBio.01908-19 (PMC6737243; doi:10.1128/mBio.01908-19)

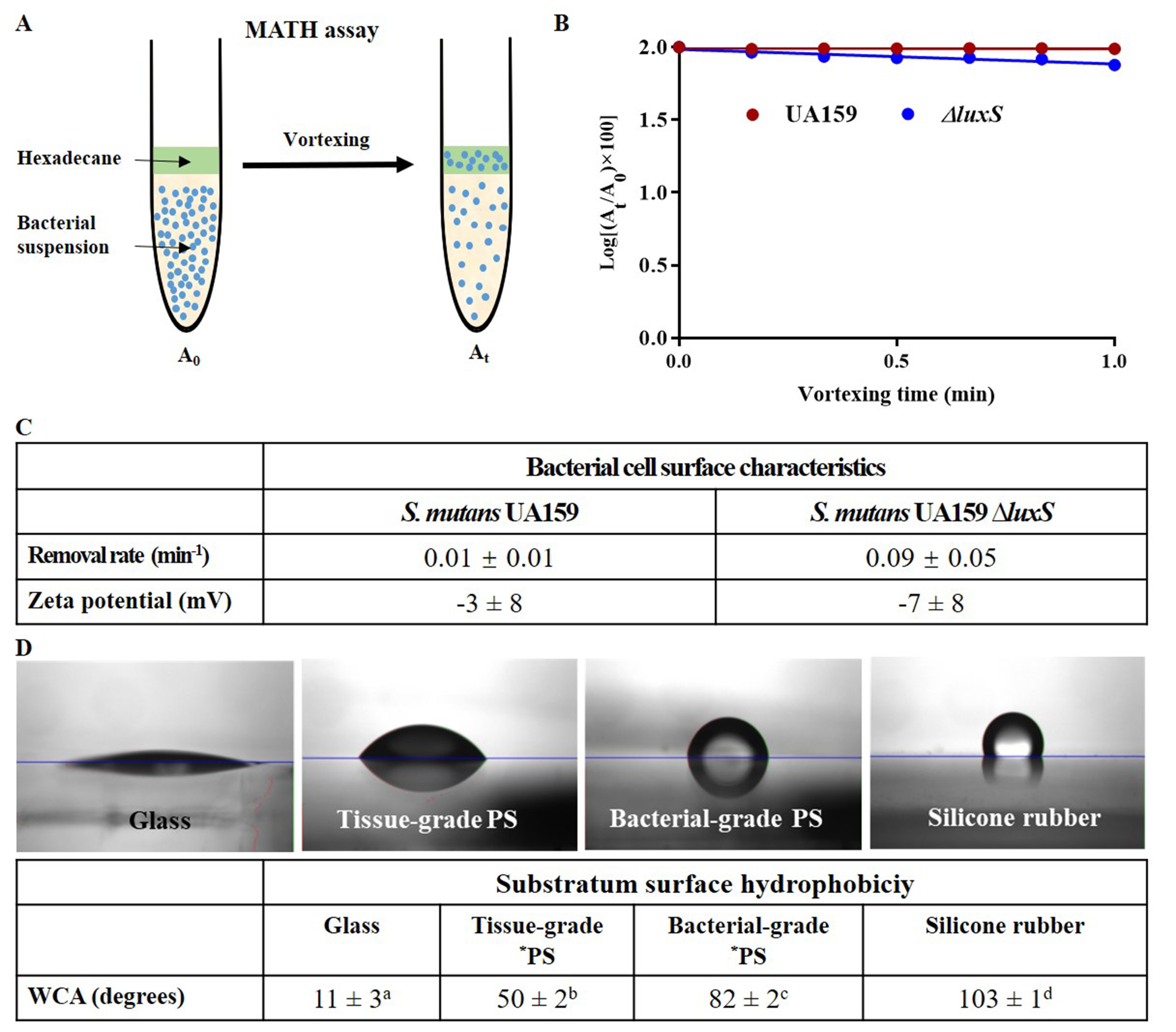

Supplement: FIG S1 [file mBio.01908-19-sf001.tif]

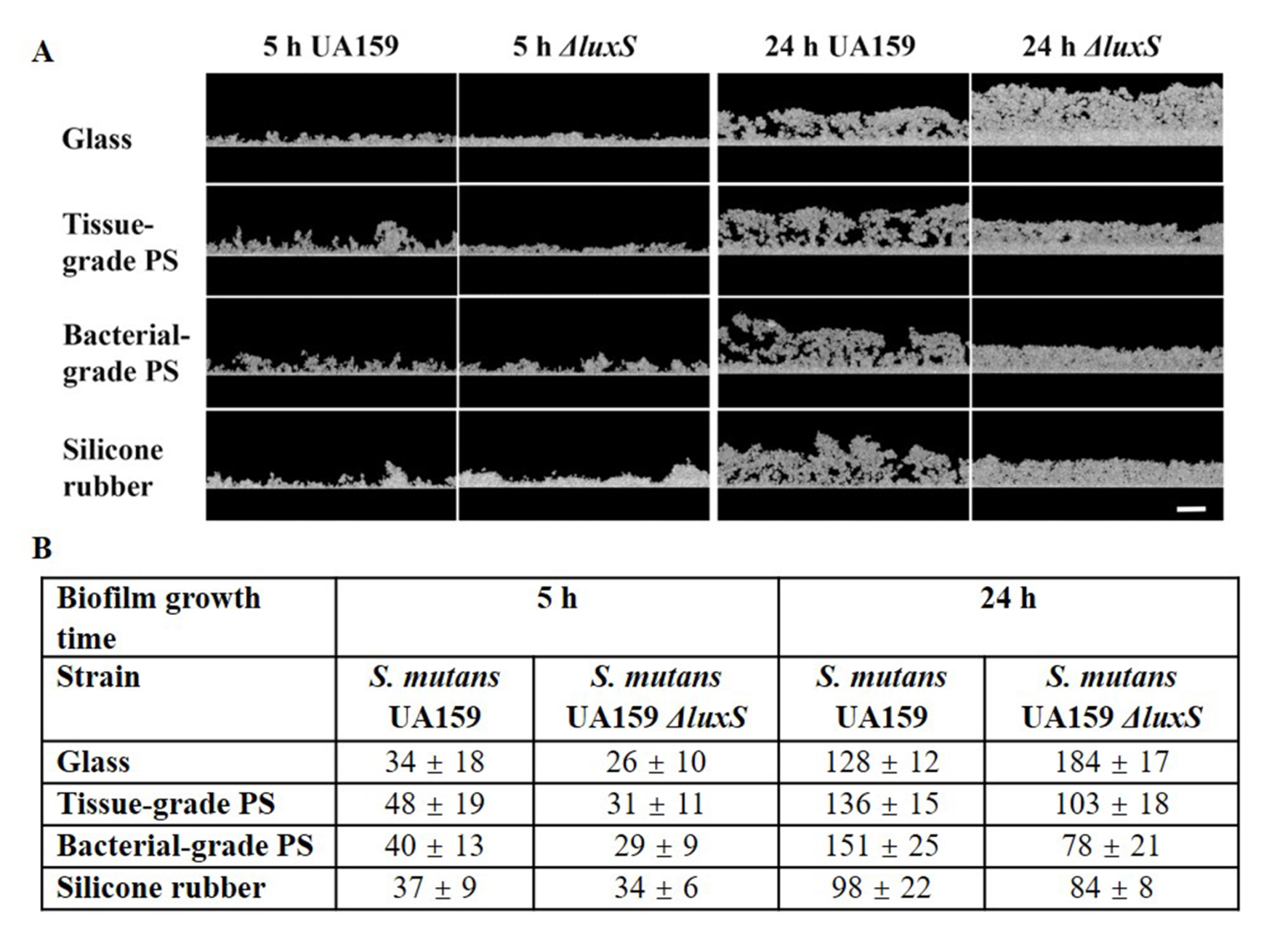

Supplement: FIG S2 [file mBio.01908-19-sf002.tif]

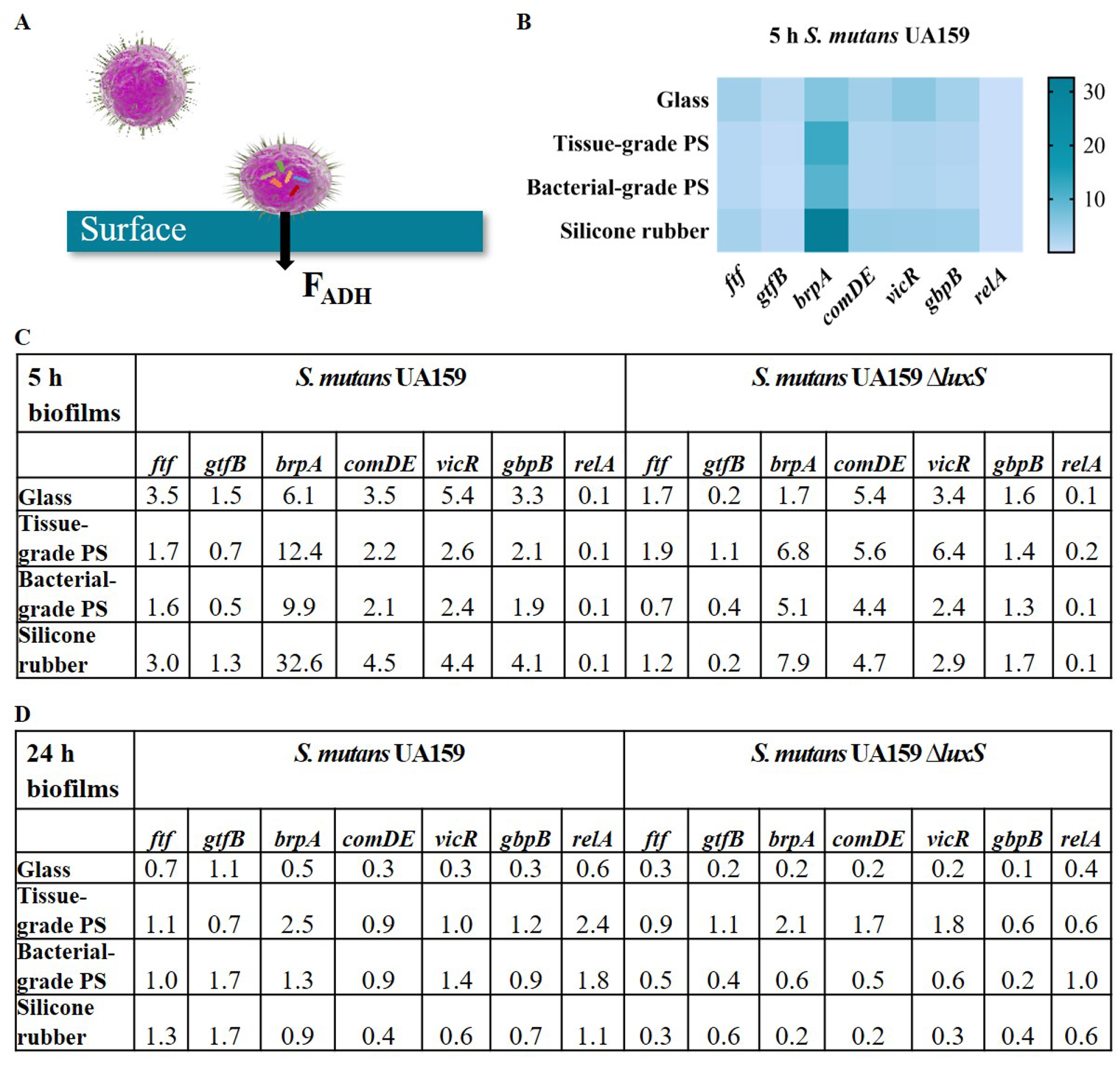

Supplement: FIG S3 [file mBio.01908-19-sf003.tif]

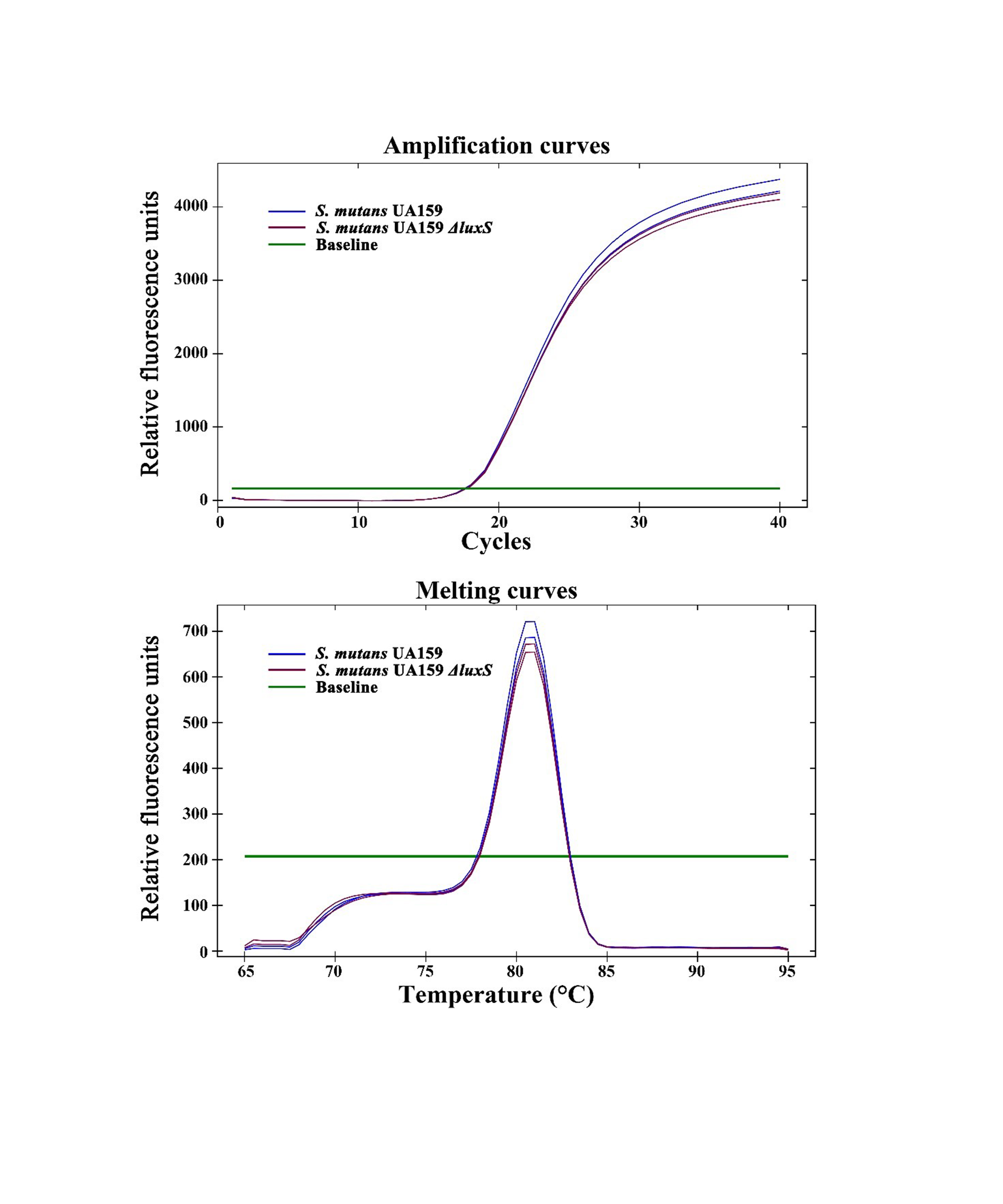

Supplement: FIG S4 [file mBio.01908-19-sf004.tif]

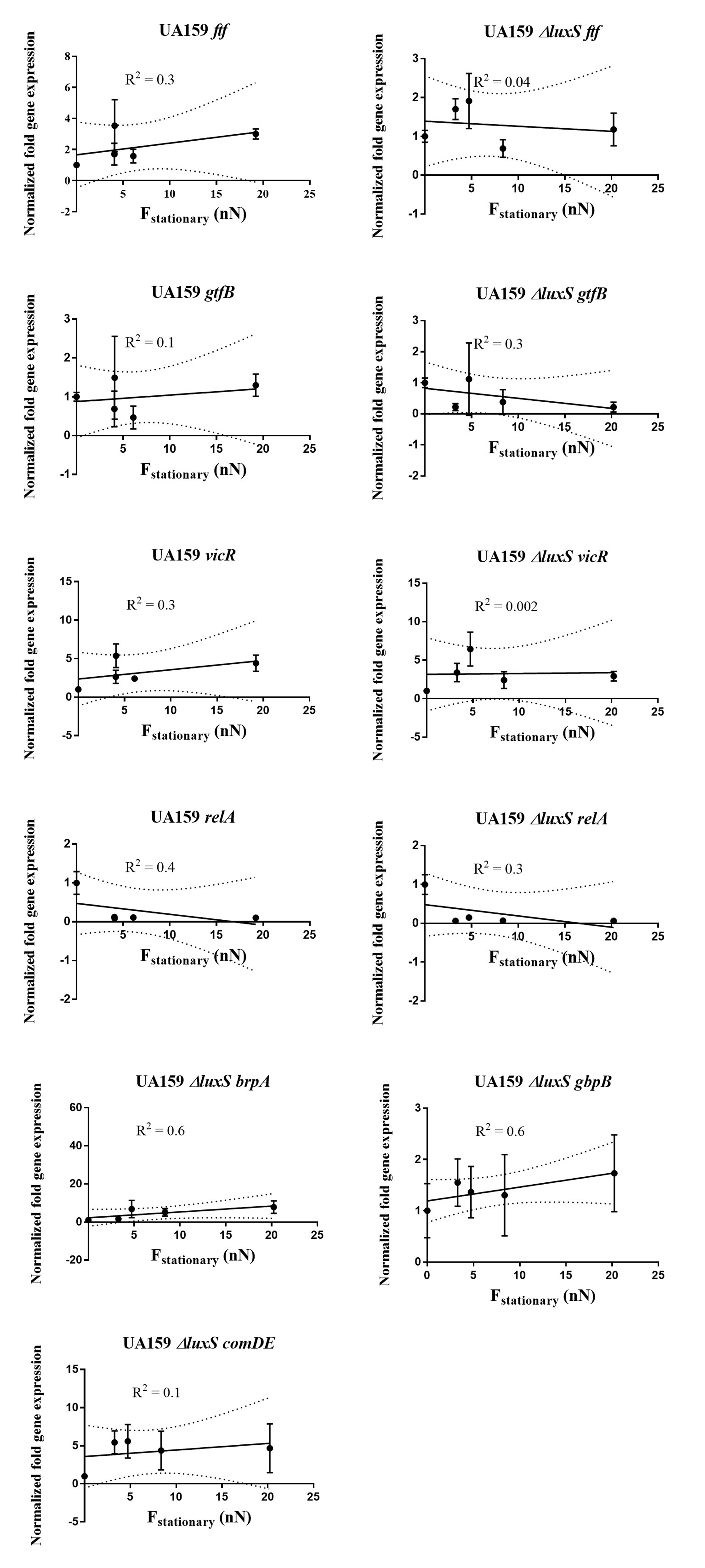

Supplement: FIG S5 [file mBio.01908-19-sf005.tif]

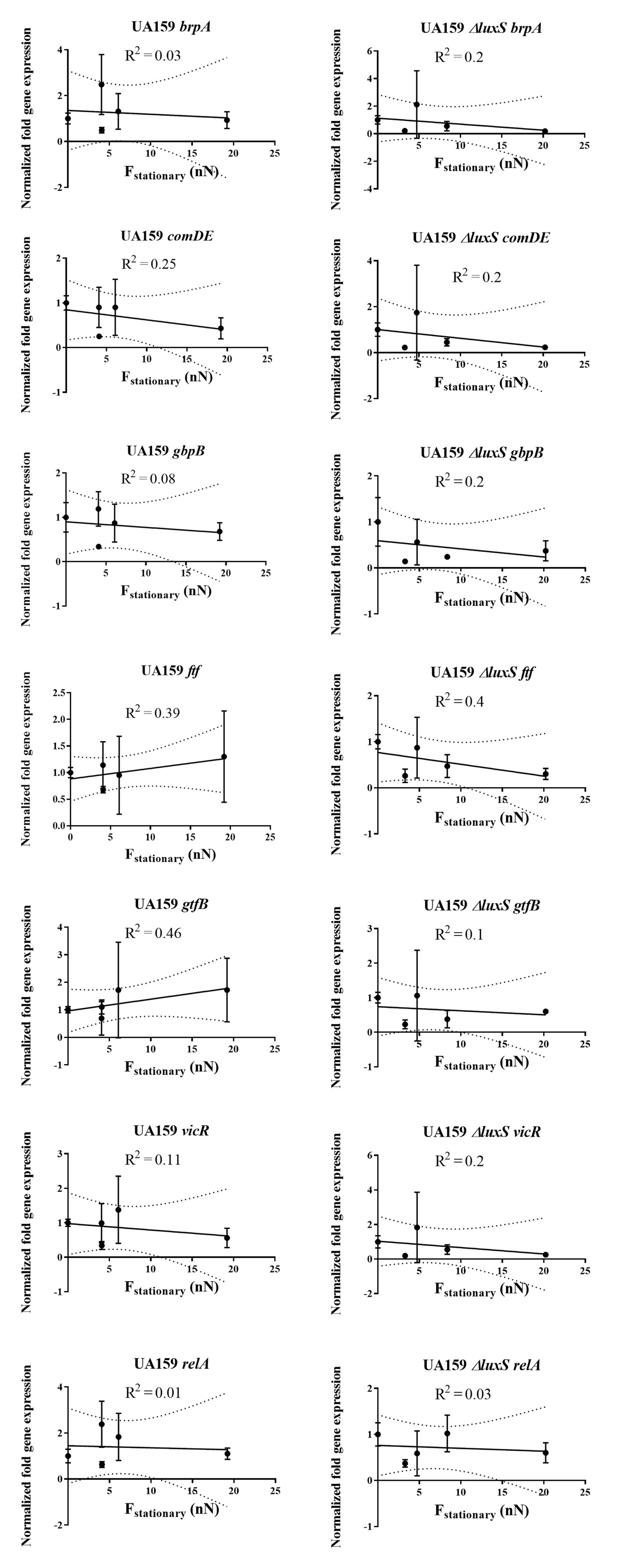

Supplement: FIG S6 [file mBio.01908-19-sf006.tif]

**Supplementary Table S1** Primer sequences for RT-qPCR used in this study.


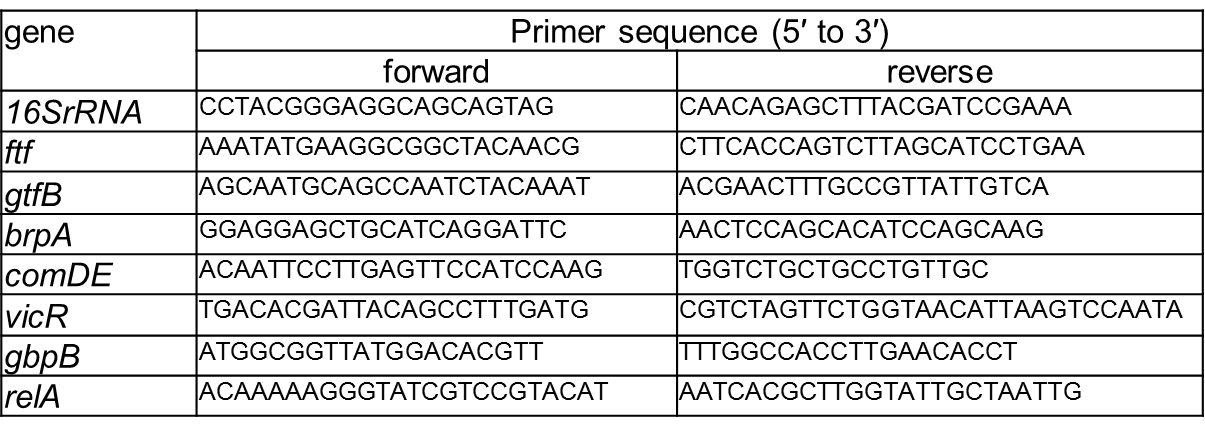

Supplement: TABLE S1 [file mBio.01908-19-st001.docx]
